# Machine Learning–Based Classification of Conduct Disorder Comorbidity in Youth With ADHD

**DOI:** 10.1002/brb3.71784

**Published:** 2026-09-23

**Authors:** Büşra Çağır, Rifat Kurban, Sevgi Özmen

**Affiliations:** ^1^ Department of Child and Adolescent Psychiatry Erciyes University Kayseri Turkey; ^2^ Department of Computer Engineering, Faculty of Engineering Abdullah Gül University Kayseri Turkey

**Keywords:** attention‐deficit/hyperactivity disorder, conduct disorder, machine learning, risk factors, support vector machine

## Abstract

**Purpose:**

To identify clinical profiles associated with conduct disorder (CD) among adolescents with attention‐deficit/hyperactivity disorder (ADHD) using integrated statistical and machine learning approaches.

**Methods:**

A total of 138 adolescents aged 11–18 years (100 ADHD; 38 ADHD+CD) were assessed using multidimensional sociodemographic and psychometric measures. Ninety‐nine variables were analyzed. Feature selection and classification analyses were performed using χ^2^ and mRMR methods combined with supervised machine learning models, including logistic regression, support vector machine (SVM), decision tree, random forest, gradient boosting, and artificial neural networks. Model performance was evaluated using accuracy, recall, specificity, and the area under the receiver operating characteristic curve (AUC).

**Results:**

Low maternal education, disciplinary punishment, school absenteeism, medication‐free periods, peer smoking and delinquency, family criminal history, physical abuse, and later ADHD diagnosis (≥12 years) consistently emerged as key factors associated with CD. In the exploratory comparison, the Top‐20 SVM had the highest observed accuracy and F1‐score among the evaluated configurations (accuracy = 0.87; AUC = 0.90; F1‐score = 0.71), with high specificity and moderate sensitivity; no single model‐selection metric was prespecified.

**Conclusion:**

These findings demonstrate that machine learning can identify clinical profiles associated with CD comorbidity in adolescents with ADHD. Such approaches may complement comprehensive clinical assessment but should not be interpreted as tools for predicting future CD development.

## Graphical abstract

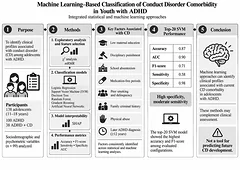

## 1 Introduction

Attention‐deficit/hyperactivity disorder (ADHD) is one of the most prevalent neurodevelopmental disorders worldwide, typically emerging in childhood and often persisting into adolescence and adulthood. Core symptoms of inattention, impulsivity, and hyperactivity are associated with marked impairments in academic, social, and occupational functioning (American Psychiatric Association 2013). ADHD is more frequently diagnosed in males than females and is characterized by substantial psychiatric comorbidity across developmental stages (Polanczyk and Rohde 2007). Among these comorbid conditions, conduct disorder (CD) represents one of the most clinically and socially consequential outcomes.

CD is defined by a persistent pattern of aggressive behavior, serious violations of rules, destruction of property, deceitfulness, and theft (American Psychiatric Association 2013). Beyond individual impairment, CD constitutes a significant public mental health concern due to its association with violence, legal problems, and long‐term psychosocial dysfunction. The disorder commonly emerges during childhood or early adolescence (Passamonti et al. 2010; Fairchild et al. 2013). Importantly, the co‐occurrence of ADHD and CD has been linked to more severe aggression, poorer treatment response, and worse long‐term prognosis compared to ADHD alone. Although CD symptoms may attenuate in some individuals over time, a considerable proportion progress to antisocial personality disorder, accompanied by elevated risks of criminal behavior and substance misuse (Loeber et al. 2000; Fergusson et al. 2005).

The developmental trajectory from ADHD to CD is widely considered multifactorial, involving interactions between biological vulnerability and environmental stressors (Beauchaine and Mcnulty 2013; Frick and Viding 2009). Emotional and physical abuse, dysfunctional parenting, prenatal tobacco exposure, low socioeconomic status, and familial criminal history have been identified as independent risk factors for CD in youth with ADHD (Danforth et al. 2016; Waschbusch 2002). Moreover, peer delinquency and adolescent substance use appear to play a critical mediating role in this transition (Marshal et al. 2003). Despite extensive research on individual predictors, the interplay between these biological, familial, and environmental variables remains complex and insufficiently modeled.

Traditional statistical approaches often rely on linear assumptions and limited variable sets, which may inadequately capture high‐dimensional interactions among risk factors. In contrast, machine learning (ML) provides a data‐driven framework capable of modeling nonlinear relationships and multidimensional predictor structures (Morgan 2005; Mcardle 2013; Dwyer et al. 2018; Koyuncugil and Özgülbaş 2009). ML methods have increasingly been applied in child and adolescent psychiatry, particularly in ADHD, autism spectrum disorder, schizophrenia, eating disorders, bipolar disorder, depression, and suicide research (Till and Briganti 2025). However, comparatively few studies have examined the prediction of CD development within ADHD populations using ML methodologies. Moreover, most available ML studies in child and adolescent psychiatry have primarily focused on diagnostic classification rather than clinically interpretable risk stratification. To date, there remains a limited number of studies integrating multidimensional clinical and sociodemographic data to identify adolescents with ADHD who are at increased risk of developing comorbid CD. This gap limits the translation of ML findings into routine clinical decision‐making and preventive mental health practice.

Within this context, the present study aimed to integrate clinical scale data and sociodemographic variables to examine factors associated with CD comorbidity in adolescents with ADHD. Beyond identifying statistically associated variables, we evaluated whether explainable ML algorithms could identify clinically interpretable patterns related to CD comorbidity. Compared with approaches examining individual predictors, this study integrates multidimensional clinical and environmental variables within a developmental psychopathology framework to provide a more comprehensive characterization of adolescents with ADHD and comorbid CD.

## 2 Methods

### 2.1 Participants

This study was conducted between August 2024 and February 2025 in the outpatient clinic of the Department of Child and Adolescent Psychiatry. Adolescents aged 11–18 years who were either newly diagnosed with ADHD or ADHD with comorbid CD according to DSM‐5 criteria, or were currently being followed with these diagnoses, were eligible for inclusion. ADHD and CD diagnoses were established by a child and adolescent psychiatrist according to DSM‐5 criteria based on comprehensive clinical evaluation, with additional support from standardized behavioral rating scales, including the Conners Parent Rating Scale (Conners 1998; Dereboy et al. 2007). Written informed consent was obtained from both participants and their parents prior to enrollment. The study protocol was approved by the Ethics Committee of … (Approval No:…).

Exclusion criteria included the presence of psychiatric or medical comorbidities other than ADHD or CD, clinically significant intellectual disability, and failure to complete assessment instruments reliably. Participants who did not provide valid or complete questionnaire data were also excluded.

Initially, 109 adolescents diagnosed with ADHD and 44 adolescents diagnosed with ADHD+CD were recruited. Among the ADHD‐only group, nine participants were excluded due to the presence of psychiatric comorbidities other than ADHD. In the ADHD+CD group, six participants were excluded: two discontinued the assessment forms before completion, two scored below the cut‐off point on the Conduct subscale of the Conners Parent Rating Scale, and two had additional psychiatric comorbidities beyond ADHD and CD.

Following the application of inclusion and exclusion criteria, the final sample consisted of 138 adolescents, including 100 with ADHD‐ only and 38 with ADHD+CD.

### 2.2 Measures

#### 2.2.1 Sociodemographic Data Collection

Study data were collected using a structured sociodemographic form developed by the authors based on a literature review and completed by the clinician. The child section of the form included age, sex, number of siblings, academic performance, and family socioeconomic status. Clinical variables recorded included age at ADHD diagnosis, current medication status, birth history, and history of disciplinary punishment.

To assess social risk factors, information was obtained regarding peer smoking, alcohol and substance use, and involvement in delinquent behaviors. The parent section included demographic information such as parental age, education, occupation, and marital status, as well as data on chronic medical conditions, regular medication use, and average household income.

#### 2.2.2 Psychometric Scales

The psychometric instruments used in this study were selected based on a developmental psychopathology framework of CD, which emphasizes the interaction between individual emotional regulation deficits, family environment, and peer‐related risk factors. Accordingly, measures were chosen to assess core domains implicated in the ADHD–CD developmental pathway, including callous–unemotional traits, empathy, resilience, attachment patterns, peer victimization, and childhood trauma exposure, as well as parental psychopathology and parenting styles. Adolescents completed validated Turkish versions of standardized instruments assessing multiple psychological domains relevant to the development of CD. These included measures of callous–unemotional traits (Inventory of Callous‐Unemotional Traits [ICU]) (Essau et al. 2006; Köse et al. 2017), attachment styles (Relationship Scales Questionnaire–Adolescent Form) (Bartholomew and Horowitz 1991; Sümer and Güngör 1999), empathic tendencies (KA‐SI Empathic Tendency Scale) (Kaya and Siyez 2010), psychological resilience (Brief Resilience Scale) (Ungar and Liebenberg 2011; Liebenberg et al. 2013; Doğan 2015), peer bullying involvement (Child and Adolescent Bullying Scale) (Vessey et al. 2019; Kirici and Ekşi 2022), and childhood trauma exposure (Childhood Trauma Questionnaire) (Bernstein et al. 1994; Şar et al. 2012).

Parents completed self‐report measures evaluating general psychopathology (Symptom Checklist‐90 [SCL‐90]) (Derogatis and Cleary 1977; Koğar 2019), affective temperament traits (TEMPS‐A) (Akiskal et al. 1998; Placidi et al. 1998; Vahip et al. 2005), ADHD symptom severity (Conners Parent Rating Scale) (Conners 1998; Dereboy et al. 2007), parenting attitudes (Karabulut Demir 2007; Şendil et al. 2023), and childhood trauma history (Bernstein et al. 1994; Şar et al. 2012).

All instruments used in the study have demonstrated acceptable psychometric properties in Turkish samples (Köse et al. 2017; Sümer and Güngör 1999; Doğan 2015; Kirici and Ekşi 2022; Şar et al. 2012; Koğar 2019; Vahip et al. 2005; Şendil et al. 2023). For the purposes of analysis, subscale scores from each instrument were entered separately into both statistical and ML models.

#### 2.2.3 Machine Learning Procedures

Machine‐learning analyses were performed in MATLAB using the Statistics and Machine Learning Toolbox, with the random‐number generator fixed at 42 for reproducibility.

For the primary analysis reported in the manuscript, chi‐square (fscchi2) and minimum‐redundancy maximum‐relevance (fscmrmr) feature rankings were calculated once using the complete dataset before cross‐validation. The mean of the two rank positions was used to define fixed Top‐10, Top‐20, Top‐30, Top‐50, and all‐99 feature sets. Because this feature selection was performed on the complete dataset before cross‐validation, the primary performance estimates may be optimistic.

Six classifiers were evaluated: lasso‐regularized logistic regression (fitclinear), polynomial‐kernel SVM (fitcsvm; internal standardization enabled), decision tree (fitctree), bagged random forest (fitcensemble; 100 learning cycles), LogitBoost gradient boosting (fitcensemble; 100 learning cycles), and a feed‐forward neural network (fitcnet; hidden layers of 10 and 5 units, 100 iterations, internal standardization enabled). No hyperparameter search was performed.

Each fixed feature‐set/model combination was evaluated using stratified five‐fold cross‐validation. Models were trained only on the training portion of each fold, and predicted labels and positive‐class scores from the five validation folds were pooled to calculate the confusion matrix, accuracy, precision, recall, F1‐score, and AUC.

Because the class distribution was imbalanced, F1‐score and recall were emphasized alongside AUC. No single model‐selection metric was prespecified; comparison of the 30 configurations was exploratory. The Top‐20 SVM was retained as the focal configuration because it had the highest observed F1‐score and accuracy, whereas the Top‐30 SVM had the highest AUC. In contrast, the separate leakage‐aware sensitivity analysis recomputed chi‐square and mRMR feature rankings using only the training data within each fold. Its results are reported in Supplementary Material S1 and do not replace the original primary analysis.

#### 2.2.4 Statistical Analysis

Statistical analyses were performed using SPSS Version 27. Continuous variables were presented as mean ± SD or median (min–max), and categorical variables as frequencies and percentages. Normality was assessed using the Kolmogorov–Smirnov and Shapiro–Wilk tests. Group comparisons were conducted using independent samples t‐tests or Mann–Whitney U tests, as appropriate. Categorical variables were compared using Pearson's chi‐square (χ^2^)test.

## 3 Results

The final sample consisted of 138 adolescents (100 ADHD‐only; 38 ADHD+CD). There were no significant differences in mean age between groups. Although the ADHD+CD group included a higher proportion of males, this difference did not reach statistical significance, and the effect size was small (Cramér's V = 0.16). (Table 1).

**TABLE 1:** Key sociodemographic and environmental characteristics by diagnostic.

Variable	ADHD *(n = 100)*	ADHD+CD *(n = 38)*	p
Age (years) *mean ± SD*	13.5 ± 1.92	13.7 ± 2.13	0.480
Sex (female/male) *n* (%)	28/72 (28%/72%)	5/33 (13.2 %/86.8%)	0.068
ADHD diagnosis ≥12 years, *n* (%)	16 (16%)	14 (36.8%)	0.008
Medication‐free treatment period, *n* (%)	55 (55%)	31 (81.5%)	0.013
Family criminal history, *n* (%)	1 (1%)	6 (15.7%)	<0.001
Peer smoking, *n* (%)	14 (14%)	16 (42.1%)	<0.001
School absenteeism, *n* (%)	12 (12%)	18 (47.3%)	<0.001
Disciplinary punishment, *n* (%)	17 (17%)	23 (60.5%)	<0.001
Maternal age (years), mean ± SD	41.9 ± 6.02	39.1 ± 6.86	0.022
Maternal education, *n* (%)			<0.001
Primary school	31 (31%)	28 (73.7%)	
High school	35 (35%)	10 (26.3%)	
University	34 (34%)	0 (0%)	
Paternal age (years), mean ± SD	45.4 ± 10.08	47.1 ± 8.24	0.586
Paternal education, *n* (%)			0.003
Primary school	34 (34%)	23 (60.5%)	
High school	28 (28%)	11 (28.8%)	
University	38 (38%)	4 (10.6%)	

Values are presented as mean ± standard deviation or n (%). Significance level set at *p* < 0.05. ADHD: attention‐deficit/hyperactivity disorder; CD: conduct disorder.

Key sociodemographic and environmental characteristics are presented in Table 1. Compared with the ADHD‐only group, adolescents with comorbid CD were more likely to have a later ADHD diagnosis (≥12 years) and to experience medication‐free treatment periods (*p* = 0.008, *p* = 0.013). Lower parental education, particularly maternal primary school education, was significantly associated with CD comorbidity (*p* = 0.003, *p* < 0.001). Family history of criminal involvement was also more frequent in the ADHD+CD group (*p* < 0.001). Peer smoking, school absenteeism, and disciplinary punishment were markedly higher among adolescents with CD (all *p* < 0.001). Overall, CD comorbidity was more strongly associated with familial, peer, and school‐related environmental factors than with basic demographic characteristics (Table 1).

### 3.1 Machine Learning Analysis

In ML analyses conducted to identify predictors of CD, χ^2^, mRMR, and SHAP methods were applied. Maternal education ranked among the top variables across all three analytical approaches. School absenteeism and disciplinary punishment were also identified among the highest‐ranking predictors in both statistical and model‐based analyses.

Peer smoking and family criminal history appeared among the top‐ranked variables in more than one method. Infant sleep problems and speech delay history were identified as prominent predictors in mRMR and SHAP analyses. Medication‐free treatment period and later ADHD diagnosis (≥12 years) were also included among the top predictors in at least one analytical approach.

**FIGURE 1:**
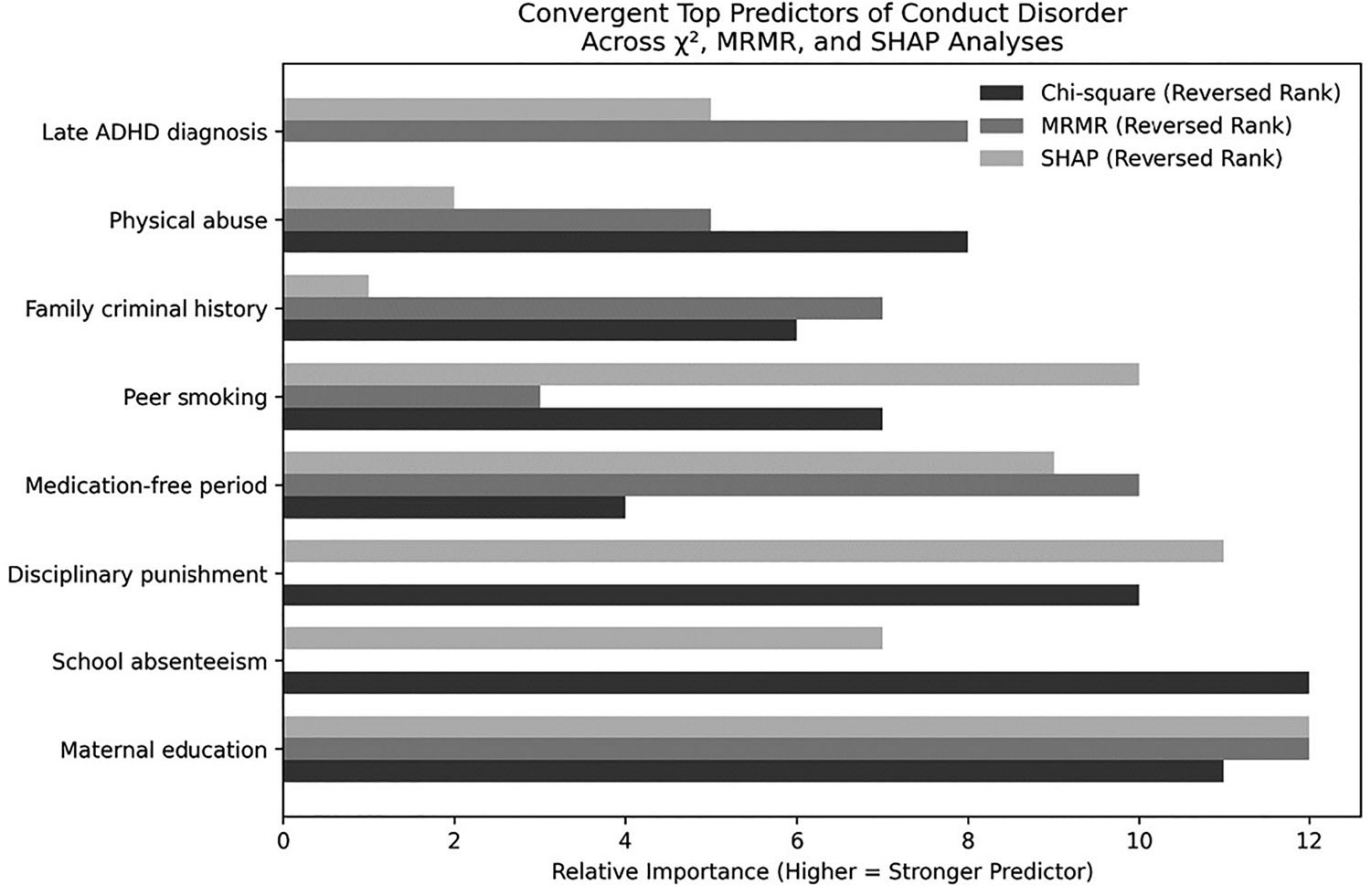
Convergent top predictors of conduct disorder identified across χ^2^, mRMR, and SHAP analyses.

The ranking positions of these variables across χ^2^, mRMR, and SHAP methods are presented in Figure 1.

### 3.2 Machine Learning Model Performance

Model performance metrics across different feature subsets are summarized in Table 2. Within this exploratory comparison, the Top‐20 SVM had the highest observed accuracy and F1‐score, whereas the Top‐30 SVM had the highest observed AUC. The Top‐20 SVM was retained only as a focal configuration for descriptive reporting; no single configuration was designated as superior because no model‐selection metric was prespecified. Models trained on smaller, high‐importance feature subsets generally demonstrated comparable or superior F1 scores compared with models including larger feature sets.

**TABLE 2:** Performance comparison of machine learning models across feature sets.

Feature set	Model	Accuracy	Precision	Recall	F1‐score	AUC
Top 10 features	Logistic regression	0.8261	0.7188	0.6053	0.6571	0.8832
Top 10 features	SVM	0.8478	0.7576	0.6579	0.7042	0.8784
Top 10 features	Decision tree	0.8188	0.7097	0.5789	0.6377	0.8145
Top 10 features	Random forest	0.8333	0.7778	0.5526	0.6462	0.9018
Top 10 features	Gradient boosting	0.8261	0.6842	0.6842	0.6842	0.8900
Top 10 features	Artificial neural network	0.8333	0.7027	0.6842	0.6933	0.8446
Top 20 features	Logistic regression	0.7899	0.6364	0.5526	0.5915	0.8482
**Top 20 features**	**SVM**	**0.8696**	**0.9167**	**0.5789**	**0.7097**	**0.8989**
Top 20 features	Decision tree	0.7609	0.5641	0.5789	0.5714	0.6757
Top 20 features	Random forest	0.7899	0.6957	0.4211	0.5246	0.8633
Top 20 features	Gradient boosting	0.7754	0.6129	0.5000	0.5507	0.8387
Top 20 features	Artificial neural network	0.7609	0.5714	0.5263	0.5479	0.7936
Top 30 features	Logistic regression	0.7826	0.6333	0.5000	0.5588	0.8482
Top 30 features	SVM	0.8261	0.9375	0.3947	0.5556	0.9150
Top 30 features	Decision tree	0.7246	0.5000	0.5789	0.5366	0.7226
Top 30 features	Random forest	0.7536	0.6111	0.2895	0.3929	0.7936
Top 30 features	Gradient boosting	0.7101	0.4667	0.3684	0.4118	0.7805
Top 30 features	Artificial neural network	0.7464	0.5429	0.5000	0.5205	0.7692
Top 50 features	Logistic regression	0.7826	0.6250	0.5263	0.5714	0.8316
Top 50 features	SVM	0.7609	0.7778	0.1842	0.2979	0.8034
Top 50 features	Decision tree	0.6594	0.3714	0.3421	0.3562	0.5988
Top 50 features	Random forest	0.7754	0.7333	0.2895	0.4151	0.8241
Top 50 features	Gradient boosting	0.8188	0.7097	0.5789	0.6377	0.8563
Top 50 features	Artificial neural network	0.8116	0.6500	0.6842	0.6667	0.8392
Top 99 features	Logistic regression	0.7754	0.6000	0.5526	0.5753	0.7437
Top 99 features	SVM	0.7464	1.0000	0.0789	0.1463	0.7650
Top 99 features	Decision tree	0.6884	0.4324	0.4211	0.4267	0.6104
Top 99 features	Random forest	0.7536	0.6667	0.2105	0.3200	0.7791
Top 99 features	Gradient boosting	0.7464	0.5517	0.4211	0.4776	0.7597
Top 99 features	Artificial neural network	0.7681	0.5938	0.5000	0.5429	0.7686

Values represent performance metrics obtained using 5‐fold cross‐validation.

**FIGURE 2:**
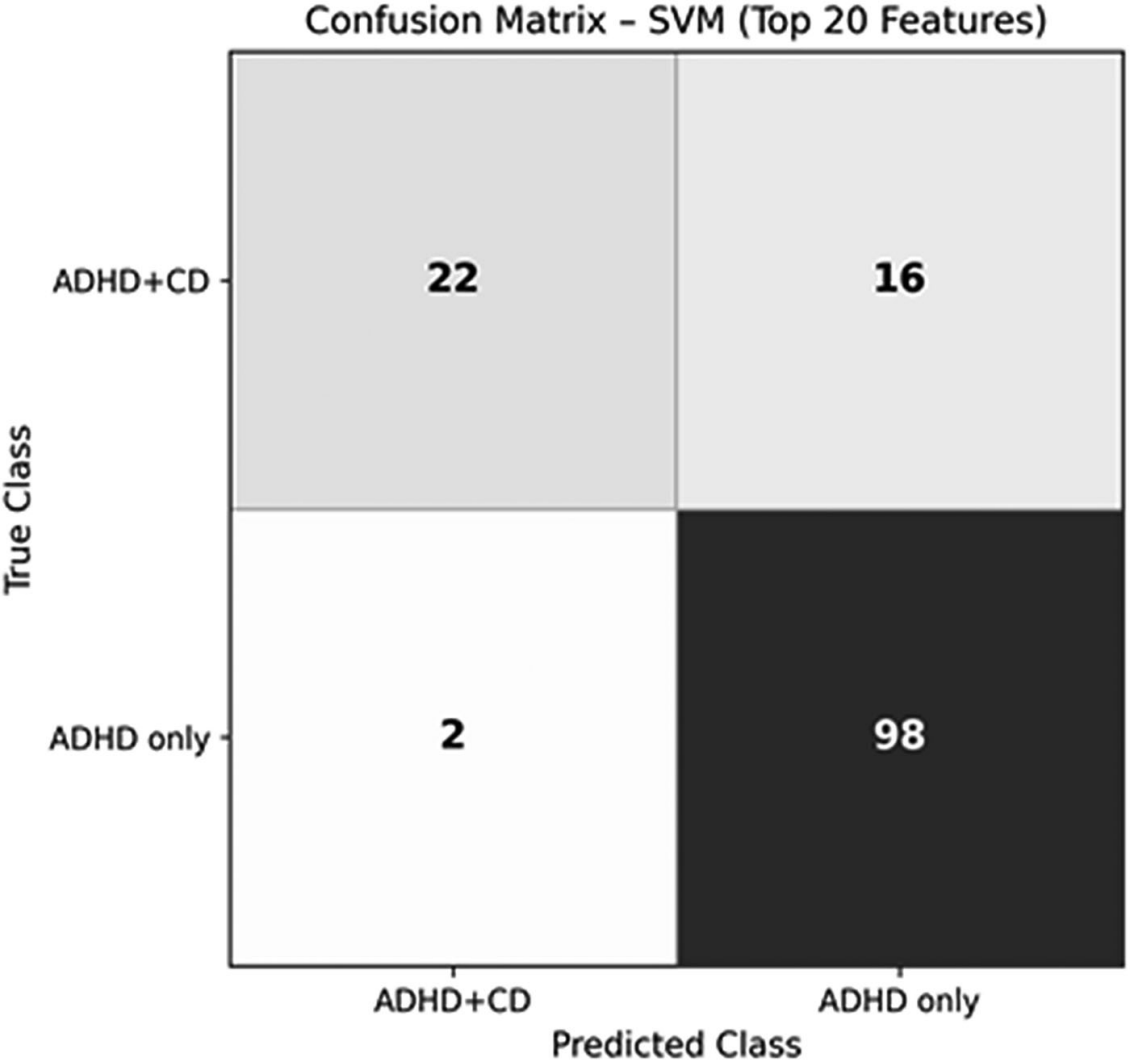
Confusion matrix for the SVM model (Top 20 features).

**FIGURE 3:**
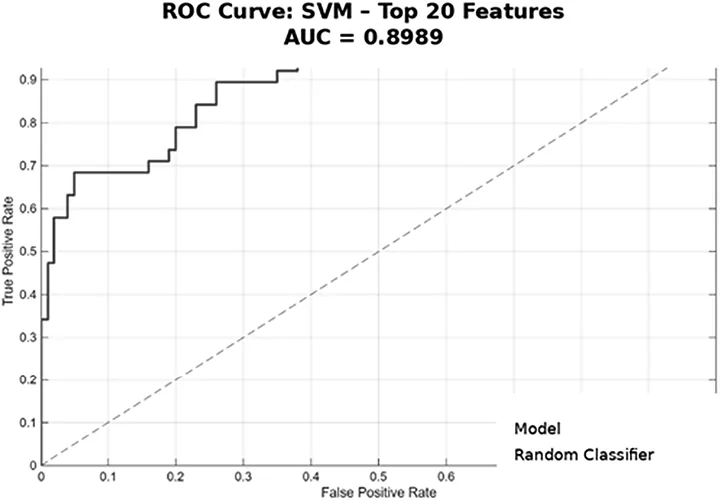
Receiver operating characteristic (ROC) curve for the SVM model (top 20 features).

The confusion matrix of the focal Top‐20 SVM model indicated high specificity (98/100 ADHD‐only cases correctly classified) and moderate sensitivity (22/38 ADHD+CD cases correctly identified) (Figure 2). The receiver operating characteristic (ROC) analysis further demonstrated strong discriminatory performance, with an AUC of 0.8989 (Figure 3). To obtain 95% confidence intervals (CIs), the 138 pooled out‐of‐fold participant records were resampled with replacement 5000 times; the true label, predicted label, and positive‐class score for each participant were resampled together. The 2.5th and 97.5th percentiles of each bootstrap distribution defined the CI limits. The CIs were narrow for specificity (0.98; 95% CI 0.95–1.00) and accuracy (0.87; 95% CI 0.81–0.92) but considerably wider for sensitivity (0.58; 95% CI 0.42–0.74) and F1‐score (0.71; 95% CI 0.56–0.83). These intervals are conditional on the pooled predictions and do not capture uncertainty arising from model refitting, feature selection, or exploratory selection among multiple configurations.

## 4 Discussion

The present study aimed to identify sociodemographic, clinical, and psychometric factors associated with CD comorbidity in adolescents with ADHD using a combination of statistical and ML approaches. Across analytical methods, several predictors consistently emerged, including low maternal education, disciplinary punishment, school absenteeism, medication‐free treatment periods, peer smoking, family history of criminal involvement, physical abuse, later age at ADHD diagnosis (≥12 years), infant sleep problems, and speech delay. These findings support the conceptualization of behavioral dysregulation as a multifactorial process shaped not only by individual vulnerability but also by familial, environmental, and social influences. Consistent with prior literature, CD is understood to arise within a developmental and epigenetic framework influenced by both genetic susceptibility and environmental exposure (Cook 2012).

Maternal education was among the most robust predictors across analyses, with higher maternal education demonstrating a protective association. Previous research has linked lower parental education to socioeconomic disadvantage, inconsistent parenting practices, and limited behavioral management strategies (Murray and Farrington 2010; Searight et al. 2001; Aydoğdu and Dilekmen 2016), all of which have been associated with increased CD comorbidity (Searight et al. 2001). These findings underscore the role of family context in shaping behavioral trajectories. Similarly, disciplinary punishment and school absenteeism emerged as strong predictors. School‐related difficulties may function both as early manifestations of conduct problems and as contextual amplifiers of behavioral escalation. Prior studies have described bidirectional relationships between disciplinary sanctions and CD, as well as associations with academic underachievement and family adversity (Hemphill et al. 2006; Kahveci 2012; Martins‐Silva et al. 2022; Lazaratou et al. 2018; Şimşek and Çöplü 2018; Karaşar and Kapçi 2016). However, these findings should be interpreted cautiously, as disciplinary punishment, school absenteeism, and peer‐related difficulties may represent not only contextual risk factors but also concurrent manifestations or consequences of CD‐related behaviors. Due to the cross‐sectional design, temporal and causal relationships between these factors and CD comorbidity cannot be established.

Treatment continuity also appeared clinically relevant. Medication‐free periods and later ADHD diagnoses were associated with higher the presence of CD in the present sample. Previous studies have linked prolonged untreated impulsivity and behavioral dysregulation to increased risk of delinquency and substance use (Thapar et al. 2001; Shaw et al. 2012; Applegate et al. 1997; Gómez‐Beneyto et al. 1994). Accordingly, delayed recognition and interruptions in treatment may represent markers of a more complex clinical course rather than direct determinants of CD development. Given that ADHD symptoms typically emerge in early childhood (Applegate et al. 1997; Gómez‐Beneyto et al. 1994), delayed diagnosis may reflect prolonged periods of unmanaged symptoms; however, causal inferences cannot be drawn from the present cross‐sectional design.

Peer‐related variables, particularly peer smoking, ranked highly across methods and emerged as important contributors in the SHAP analysis. This aligns with research emphasizing the growing influence of peer affiliation during adolescence and the role of deviant peer networks in the development of conduct problems (Siyez and Aysan 2007; Muuss 1971). Likewise, family criminal history and physical abuse were significant predictors, consistent with evidence linking intergenerational risk and maltreatment to increased aggression and reduced empathy—core features of CD (Gómez‐Beneyto et al. 1994; Kim‐Cohen et al. 2005; Arman 2019; Bauer et al. 2022). Infant sleep problems and speech delay history, identified as important predictors, may reflect early developmental vulnerabilities associated with later self‐regulatory difficulties and externalizing behaviors. Previous studies have also reported that early sleep disturbances and language development delays are associated with later emotional and behavioral problems (Lam and Lam 2021; Bishop et al. 2016).

Among the evaluated ML algorithms, the SVM model provided quantitative support for the proposed risk profiling framework. SVM models are particularly suited to high‐dimensional psychiatric datasets and have demonstrated favorable performance in previous psychiatric prediction studies (Till and Briganti 2025). In the exploratory comparison, the Top‐20 SVM had the highest observed accuracy and F1‐score; however, no single configuration was designated as superior because no model‐selection metric was prespecified. Models trained on smaller, information‐rich feature sets generally outperformed those including all variables, underscoring the importance of feature selection in psychiatric prediction modeling.

The high specificity observed in the SVM model indicates good discrimination of ADHD‐only cases; however, the moderate sensitivity suggests that a substantial proportion of ADHD+CD cases may remain unidentified. Therefore, the model should not be considered a stand‐alone screening or diagnostic tool but rather a complementary approach to support comprehensive clinical assessment. To address concerns regarding the “black box” nature of ML methods, SHAP analysis was applied to quantify the relative contribution of individual predictors. This approach enhanced model transparency and facilitated clinical interpretability. The convergence of χ^2^, mRMR, and SHAP findings further supports the consistency of the identified feature pattern. Because the two diagnostic groups were imbalanced (100 vs. 38), overall accuracy was strongly influenced by correct classification of the majority ADHD‐only group and may overestimate model performance. Therefore, model performance should be interpreted primarily through class‐sensitive metrics (sensitivity, F1‐score, and AUC) together with the confusion matrix, rather than through accuracy alone.

From a clinical standpoint, disciplinary punishment, school absenteeism, peer‐related factors, treatment adherence, and parental educational level represent observable indicators that may support comprehensive assessment and structured monitoring of adolescents with ADHD in outpatient care. The identification of potentially modifiable contextual factors may inform future risk assessment and prevention strategies. Adolescents presenting these clinical profiles may require closer clinical monitoring and may represent a target population for future parent‐focused and school‐based intervention studies. Machine learning–supported profiling should complement—rather than replace—clinical judgment within a stepped‐care model of ADHD management. However, ML‐based risk profiling remains susceptible to misclassification and should be interpreted within the broader clinical context rather than used as a stand‐alone decision tool.

The study's strengths include the use of a multidimensional dataset, comparison of multiple ML algorithms, and incorporation of explainability analyses. However, the single‐center design, which may have limited the sociodemographic diversity of the study population, together with the modest sample size and lack of external validation, limits the generalizability of the findings. In particular, the small number of ADHD+CD cases (n = 38; approximately seven to eight per cross‐validation fold) may have reduced the stability of minority‐class performance estimates, as reflected by the wide confidence interval for sensitivity. Furthermore, given that 99 candidate variables and 30 machine‐learning model configurations were evaluated, the reported performance estimates should be interpreted cautiously and considered preliminary, as they may be subject to optimism. Specifically, because feature selection in the original primary analysis was performed on the complete dataset before cross‐validation, the primary performance estimates may be optimistic. The leakage‐aware sensitivity analysis reported in Supplementary Material S1 repeated feature selection within each training fold and is presented separately from, rather than as a replacement for, the original primary analysis. Although cross‐validation procedures were applied to reduce overfitting, external validation in larger, independent multicenter samples is required to confirm the robustness and generalizability of these findings. Because the same cross‐validation results were used both to compare the 30 configurations and to report the focal configuration's performance, and no independent outer cross‐validation loop was used, model‐selection optimism may remain. The pooled‐prediction bootstrap intervals likewise do not represent all uncertainty associated with model fitting, pre‐cross‐validation feature selection, or configuration selection. Furthermore, the cross‐sectional design precludes causal inference regarding the progression from ADHD to CD. In conclusion, integrating statistical analyses with ML provides a structured and interpretable framework for identifying clinical profiles associated with CD comorbidity among adolescents with ADHD, while reinforcing the central role of comprehensive clinical assessment.

## Author Contributions

**Büşra Çağır**: conceptualization, methodology, data curation, investigation, formal analysis, funding acquisition, resources, writing – original draft. **Rifat Kurban**: methodology, software, validation, formal analysis, supervision, writing – review and editing. **Sevgi Özmen**: conceptualization, methodology, data curation, investigation, supervision, funding acquisition, visualization, project administration, resources, writing – original draft, writing – review and editing.

## Funding

The authors have nothing to report.

## Ethics Statement

The study was approved by the Ethics Committee of Erciyes University Faculty of Medicine (Decision No: 2024/218).

## Conflicts of Interest

The authors declare no conflicts of interest.

## Supporting information




**Supplementary Material**: brb371784‐sup‐0001‐SuppMat.docx

## Data Availability

The datasets generated and analyzed during the current study are available from the corresponding author upon reasonable request and subject to ethical approval.
