# Supplementary material for: Machine Learning–Based Classification of Conduct Disorder Comorbidity in Youth With ADHD
Source: Brain Behav. 2026 Sep 23;16(9):e71784. doi: 10.1002/brb3.71784 (PMC13601744; doi:10.1002/brb3.71784)
Supplement: Supplementary file 1 [file BRB3-16-e71784-s001.docx]

**Supplementary Material S1**

**Within-Fold Feature-Selection Sensitivity Analysis**

*Machine Learning–Based Classification of Conduct Disorder Comorbidity in Youth with ADHD*

# S1. Purpose

This supplementary sensitivity analysis evaluated the same dataset, feature-set sizes, and six classifier families as the primary analysis while repeating feature selection independently within each training fold. The analysis is reported separately and does not replace the primary pre-cross-validation feature-ranking results in the manuscript.

# S2. Methods

## S2.1 Data and cross-validation

The analysis included 138 participants (100 ADHD-only and 38 ADHD+CD) and 99 candidate predictors. A fixed random seed of 42 was used. One stratified five-fold partition was generated and reused across all 30 feature-set/model configurations. Fold sizes were 27, 28, 28, 28, and 27 participants; both diagnostic classes were represented in every fold.

## S2.2 Within-fold feature selection

For each fold, chi-square (fscchi2) and minimum-redundancy maximum-relevance (fscmrmr) rankings were calculated using only the training observations. The two rank positions were averaged, and fold-specific Top-10, Top-20, Top-30, and Top-50 subsets were constructed. The all-99 condition included every predictor. The validation observations were not used to form the feature rankings.

## S2.3 Model fitting and pooled predictions

The six classifiers and model specifications matched the primary analysis: lasso-regularized logistic regression, polynomial-kernel SVM, decision tree, bagged random forest with 100 learning cycles, LogitBoost gradient boosting with 100 learning cycles, and a neural network with hidden layers of 10 and 5 units and an iteration limit of 100. SVM and neural-network standardization was performed internally using the corresponding MATLAB model options. Models were fitted on each training fold and evaluated on its held-out validation fold. The predicted labels and positive-class scores from the five validation folds were pooled to calculate accuracy, precision, recall, specificity, F1-score, balanced accuracy, AUC, and confusion-matrix counts.

## S2.4 Bootstrap confidence intervals

For the primary focal Top-20 SVM and the within-fold configuration with the highest observed F1-score and AUC (Top-10 Random Forest), 95% confidence intervals were calculated from 5,000 participant-level bootstrap resamples of the pooled out-of-fold records. The true label, predicted label, and positive-class score were resampled together. The 2.5th and 97.5th percentiles defined the interval limits.

# S3. Results

Across the 30 within-fold configurations, the highest observed accuracy was 0.826 for the Top-20 SVM. The highest observed F1-score (0.615) and AUC (0.840) occurred for the Top-10 Random Forest. The Top-20 SVM yielded precision 0.850, recall 0.447, specificity 0.970, F1-score 0.586, and AUC 0.817. Absolute differences of at least 0.05 relative to the primary results were observed in 15/30 configurations for F1-score and 15/30 configurations for AUC (Figure S1).

For the Top-20 condition, 36 different predictors were selected across the five folds, and 10 predictors were selected in every fold. The mean pairwise Jaccard similarity was 0.536. The complete performance results, bootstrap intervals, and feature-set stability summaries are reported in Tables S1–S3; Top-20 selection frequencies are shown in Figure S2.

*Figure S1. Differences in F1-score and AUC between the within-fold sensitivity analysis and the primary analysis across all 30 configurations.*

*Figure S2. Feature-selection frequency for the Top-20 within-fold condition. Dark gray denotes features also included in the primary global Top-20 set.*

*Figure S3. Primary and within-fold sensitivity estimates for the Top-20 SVM.*

# Table S1. Within-fold performance across all 30 configurations

| **Feature set** | **Model** | **Accuracy** | **Precision** | **Recall** | **Specificity** | **F1** | **Balanced accuracy** | **AUC** |
| --- | --- | --- | --- | --- | --- | --- | --- | --- |
| Top 10 | Artificial Neural Network | 0.775 | 0.606 | 0.526 | 0.870 | 0.563 | 0.698 | 0.751 |
| Top 10 | Decision Tree | 0.739 | 0.526 | 0.526 | 0.820 | 0.526 | 0.673 | 0.753 |
| Top 10 | Gradient Boosting | 0.775 | 0.600 | 0.553 | 0.860 | 0.575 | 0.706 | 0.794 |
| Top 10 | Logistic Regression | 0.783 | 0.633 | 0.500 | 0.890 | 0.559 | 0.695 | 0.833 |
| Top 10 | Random Forest | 0.819 | 0.741 | 0.526 | 0.930 | 0.615 | 0.728 | 0.840 |
| Top 10 | SVM | 0.797 | 0.679 | 0.500 | 0.910 | 0.576 | 0.705 | 0.816 |
| Top 20 | Artificial Neural Network | 0.775 | 0.600 | 0.553 | 0.860 | 0.575 | 0.706 | 0.733 |
| Top 20 | Decision Tree | 0.739 | 0.525 | 0.553 | 0.810 | 0.538 | 0.681 | 0.740 |
| Top 20 | Gradient Boosting | 0.797 | 0.708 | 0.447 | 0.930 | 0.548 | 0.689 | 0.792 |
| Top 20 | Logistic Regression | 0.761 | 0.593 | 0.421 | 0.890 | 0.492 | 0.656 | 0.814 |
| Top 20 | Random Forest | 0.797 | 0.708 | 0.447 | 0.930 | 0.548 | 0.689 | 0.802 |
| Top 20 | SVM | 0.826 | 0.850 | 0.447 | 0.970 | 0.586 | 0.709 | 0.817 |
| Top 30 | Artificial Neural Network | 0.775 | 0.606 | 0.526 | 0.870 | 0.563 | 0.698 | 0.791 |
| Top 30 | Decision Tree | 0.681 | 0.417 | 0.395 | 0.790 | 0.405 | 0.592 | 0.637 |
| Top 30 | Gradient Boosting | 0.739 | 0.545 | 0.316 | 0.900 | 0.400 | 0.608 | 0.758 |
| Top 30 | Logistic Regression | 0.732 | 0.520 | 0.342 | 0.880 | 0.413 | 0.611 | 0.786 |
| Top 30 | Random Forest | 0.768 | 0.636 | 0.368 | 0.920 | 0.467 | 0.644 | 0.794 |
| Top 30 | SVM | 0.761 | 0.692 | 0.237 | 0.960 | 0.353 | 0.598 | 0.813 |
| Top 50 | Artificial Neural Network | 0.732 | 0.512 | 0.579 | 0.790 | 0.543 | 0.684 | 0.737 |
| Top 50 | Decision Tree | 0.681 | 0.429 | 0.474 | 0.760 | 0.450 | 0.617 | 0.636 |
| Top 50 | Gradient Boosting | 0.754 | 0.567 | 0.447 | 0.870 | 0.500 | 0.659 | 0.802 |
| Top 50 | Logistic Regression | 0.761 | 0.576 | 0.500 | 0.860 | 0.535 | 0.680 | 0.802 |
| Top 50 | Random Forest | 0.775 | 0.706 | 0.316 | 0.950 | 0.436 | 0.633 | 0.781 |
| Top 50 | SVM | 0.761 | 0.857 | 0.158 | 0.990 | 0.267 | 0.574 | 0.839 |
| Top 99 | Artificial Neural Network | 0.739 | 0.524 | 0.579 | 0.800 | 0.550 | 0.689 | 0.749 |
| Top 99 | Decision Tree | 0.674 | 0.405 | 0.395 | 0.780 | 0.400 | 0.587 | 0.605 |
| Top 99 | Gradient Boosting | 0.790 | 0.737 | 0.368 | 0.950 | 0.491 | 0.659 | 0.776 |
| Top 99 | Logistic Regression | 0.717 | 0.487 | 0.500 | 0.800 | 0.494 | 0.650 | 0.772 |
| Top 99 | Random Forest | 0.768 | 0.800 | 0.211 | 0.980 | 0.333 | 0.595 | 0.743 |
| Top 99 | SVM | 0.754 | 1.000 | 0.105 | 1.000 | 0.190 | 0.553 | 0.776 |

# Table S2. Participant-level bootstrap confidence intervals

| **Configuration** | **Metric** | **Estimate** | **95% CI** |
| --- | --- | --- | --- |
| Top 20 SVM | Accuracy | 0.826 | 0.761–0.884 |
| Top 20 SVM | Recall | 0.447 | 0.286–0.610 |
| Top 20 SVM | Specificity | 0.970 | 0.933–1.000 |
| Top 20 SVM | Precision | 0.850 | 0.680–1.000 |
| Top 20 SVM | F1 | 0.586 | 0.413–0.724 |
| Top 20 SVM | BalancedAccuracy | 0.709 | 0.626–0.791 |
| Top 20 SVM | AUC | 0.817 | 0.725–0.896 |
| Top 10 Random Forest | Accuracy | 0.819 | 0.754–0.877 |
| Top 10 Random Forest | Recall | 0.526 | 0.369–0.679 |
| Top 10 Random Forest | Specificity | 0.930 | 0.877–0.973 |
| Top 10 Random Forest | Precision | 0.741 | 0.565–0.900 |
| Top 10 Random Forest | F1 | 0.615 | 0.462–0.741 |
| Top 10 Random Forest | BalancedAccuracy | 0.728 | 0.643–0.810 |
| Top 10 Random Forest | AUC | 0.840 | 0.761–0.907 |

# Table S3. Feature-set stability across folds

| **Feature set** | **Union** | **Selected in all 5** | **Mean Jaccard** | **Min Jaccard** | **Max Jaccard** | **Mean overlap with primary set** |
| --- | --- | --- | --- | --- | --- | --- |
| Top 10 | 16 | 4 | 0.559 | 0.429 | 0.667 | 0.780 |
| Top 20 | 36 | 10 | 0.536 | 0.429 | 0.667 | 0.750 |
| Top 30 | 53 | 15 | 0.536 | 0.463 | 0.622 | 0.800 |
| Top 50 | 78 | 24 | 0.574 | 0.493 | 0.667 | 0.808 |
| Top 99 | 99 | 99 | 1.000 | 1.000 | 1.000 | 1.000 |
